# Genetic perturbation of IL-6 receptor signaling pathway and risk of multiple respiratory diseases

**DOI:** 10.1186/s12967-024-05366-6

**Published:** 2024-06-19

**Authors:** Dongsheng Wu, Zhipeng Gong, Xiaohu Hao, Lunxu Liu

**Affiliations:** Department of Thoracic Surgery and Institute of Thoracic Oncology, West China Hospital, Sichuan University, No. 37, Guoxue Alley, Chengdu, 610041 Sichuan China

**Keywords:** Interleukin-6, Respiratory diseases, Therapeutic target, Mendelian randomization

## Abstract

Dysregulation of inflammation can lead to multiple chronic respiratory diseases, such as chronic obstructive pulmonary disease (COPD) and asthma. Interleukin-6 (IL6) is crucial in regulating the inflammatory cascade, but the causal link between IL6 signaling downregulation and respiratory diseases risk is unclear. This study uses Mendelian randomization to examine the effects of IL6R blockade on respiratory diseases. Analyzing data from 522,681 Europeans, 26 genetic variants were obtained to mimic IL6R inhibition. Our findings show that IL6R blockade significantly reduces the risk of COPD (OR = 0.71, 95% CI = 0.60–9.84) and asthma (OR = 0.82, 95% CI = 0.74–0.90), with protective trends for bronchitis, pulmonary embolism, and lung cancer. Results were consistent across methods, with no significant heterogeneity or pleiotropy. These insights suggest IL6R downregulation as a potential therapeutic target for respiratory diseases, meriting further clinical investigation.

**Supplementary Information:**

The online version contains supplementary material available at 10.1186/s12967-024-05366-6.

To the Editor,

Dysregulation of immune responses can result in uncontrolled inflammation, which may contribute to the onset of chronic respiratory diseases such as chronic obstructive pulmonary disease (COPD), pulmonary fibrosis, asthma, and lung cancer [1]. Interleukin-6 (IL6), an inflammatory cytokine, plays a central role in regulating the inflammatory cascade [2]. Recent research has demonstrated the potential benefits of inhibiting the IL6 receptor (IL6R) in improving COVID-19 outcomes [3]. However, the causal relationship between the downregulation of the IL6 signaling pathway and the risk of multiple respiratory diseases remains uncertain.

Mendelian randomization (MR) studies offer valuable insights into estimating potential effects in clinical intervention trials. Genetic variants serving as proxies for IL6R perturbation can be utilized as instrumental variables (IVs) within the MR framework to explore corresponding drug effects. Thus, a MR study was conducted to explore the potential effect of IL6R blockade on risk of respiratory diseases.

A genetic instrument comprising 26 genetic variants located within IL6R (r^2^ < 0.1, window size = 300 kb) was derived from a meta-analysis of high-sensitivity C-reactive protein (CRP) genome-wide association study (GWAS) data involving 522,681 European individuals (Table S1) [4]. These genetic variants serve to emulate the therapeutic inhibition of IL6R (e.g., tocilizumab), which downregulates IL6 signaling by inhibiting both classic and trans signaling pathways (Fig. 1). Our investigation focused on assessing the impact of these IL6R variants on the risk of multiple respiratory diseases, including COPD, bronchitis, asthma, pulmonary embolism, idiopathic pulmonary fibrosis, and lung cancer (Table 1). The ethical approval for this study can be found from the corresponding studies, as only summary-level data was analyzed.

**Fig. 1:**
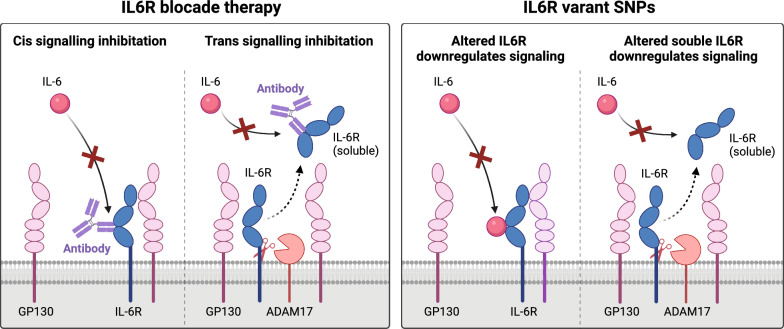
A schematic diagram depicting the effects of IL6R antibody and IL6R genetic instruments on the IL6 signaling pathway (created with BioRender.com). The left section of the diagram represents the impact of IL6R monoclonal antibody on the IL6 signaling pathway. The right section illustrates the use of IL6R genetic instruments for IL6R blockade to act as a proxy for intervention. *GP130* glycoprotein 130, *IL6R* interleukin 6 receptor, *ADAM17* a disintegrin and metalloprotease 17, *SNP* single nucleotide polymorphism

**Table 1:** Data source for IL6R blockade and respiratory diseases

Phenotypes	Case/control	Consortium	Population	Year	Data source
Interleukin 6 blockade	522,681	CHARGE and UKbiobank	European	2022	PMID: 34927447
COPD	18,266/311,286	FinnGen	European	2022	www.finngen.fi/en
Bronchitis	55,222/322,055	FinnGen	European	2022	www.finngen.fi/en
Asthma	56,167/352,255	UKbiobank	European	2021	PMID: 34103634
Pulmonary embolism	9243/367,108	FinnGen	European	2022	www.finngen.fi/en
Idiopathic pulmonary fibrosis	4125/20,464	–	European	2021	PMID: 35688625
Lung cancer	29,266/56,450	ILCCO	European	2017	PMID: 28604730

*COPD* chronic obstructive pulmonary disease, *CHARGE* Cohorts for Heart and Aging Research in Genomic Epidemiology, *ILCCO* International Lung Cancer Consortium

In this study, the main casual estimates utilized the inverse-variance weighted (IVW) method, complemented by a secondary analysis utilizing the weighted median and MR Egger methods. Heterogeneity was assessed through the P value of Cochran’s Q test. The evaluation of horizontal pleiotropy was conducted using the MR pleiotropy residual sum and outlier (MR-PRESSO) approach, followed by a recalculation of the causal effect after removing outliers. A funnel plot was used to detect heterogeneity. Furthermore, the leave-one-out method was used to calculate the combined effect of each remaining SNP. All MR analyses were performed using the “TwoSampleMR” and “MRPRESSO” packages in R software (version 4.3.2).

The selected genetic instrument accounted for 1.14% of the variance in IL6 signaling downregulation. All F-statistics were all above 10, which suggested a sufficient strength (Table S1). Our results showed that genetically proxied IL6R blockade was significantly related with a reduced risk of COPD (OR_IVW_ = 0.71, 95% CI = 0.60–9.84) and asthma (OR_IVW_ = 0.82, 95% CI = 0.74 − 0.90) (Fig. 2). For other respiratory diseases, downregulation of IL6 signaling pathway presented a protective trend for bronchitis (OR_IVW_ = 0.95, 95% CI = 0.87–1.05), pulmonary embolism (OR_IVW_ = 0.90, 95% CI = 0.73–1.11), and lung cancer (OR_IVW_ = 0.87, 95% CI = 0.73–1.04), but not for idiopathic pulmonary fibrosis (OR_IVW_ = 1.27, 95% CI = 0.87–1.85). Furthermore, both the weighted median and MR-Egger approaches exhibited similar trends. No significant heterogeneity or pleiotropy was detected. Additionally, employing MR-PRESSO revealed no outlier SNPs, suggesting minimal horizontal pleiotropy. Scatter plots, funnel plots, and leave-one-out analyses are depicted in Figures S1–3.

**Fig. 2:**
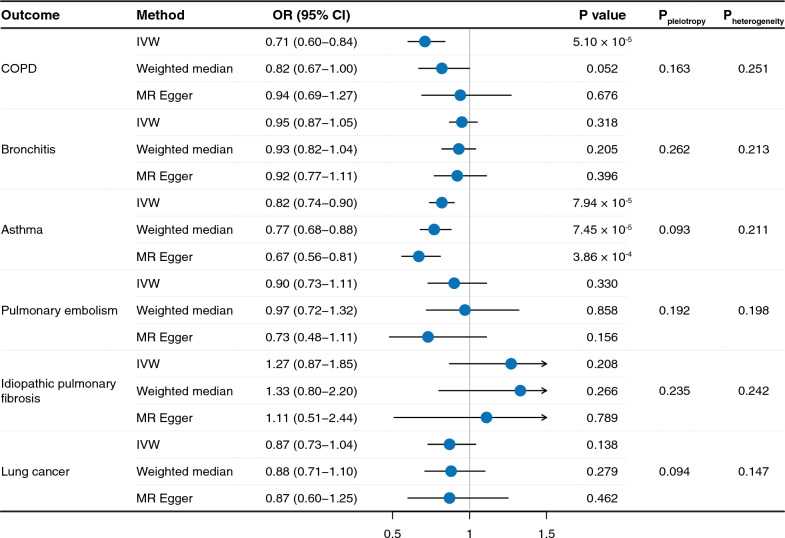
MR estimates of IL6R blockade and multiple respiratory diseases. *OR* odds ratio, *CI* confidence interval, *IVW* inverse variance weighted, *COPD* chronic obstructive pulmonary disease

Utilizing 26 genetic instruments for IL6R blockade, this study offers evidence suggesting that downregulation of IL6 signaling pathways may confer protective effect against the development of COPD and asthma. It is worth to note that these genetic instruments have shown similar effects to pharmacological IL6R blockade, correlating with alterations in concentrations of CRP, fibrinogen, circulating IL-6, and soluble IL6R [4]. Using these genetic instruments, Hamilton et al. recently reported a protective effect of downregulation of IL6 signaling pathways on COVID-19 outcomes [5]. Thus, further positive control analysis was not conducted in this study. Nevertheless, several limitations should be noted. Firstly, IL-6 signaling is intricate, comprising both classical and trans signaling components, and dissecting these sub-pathways surpasses the limitations of MR. Secondly, proxying IL6R blockade was only feasible at the IL6R locus. However, previous genetic associations at this locus have concurred with observed effects of IL6 blockers (e.g., tocilizumab), supporting our assertion that variants at the locus can act as proxies for IL6R blockade. Finally, the extent of variance in CRP concentrations explained by the 26 variants is relatively small. In summary, this study provides valuable insights into the causal role of IL6 signaling downregulation in risk of respiratory diseases, especially COPD and asthma. Targeting IL6R may serve as an actionable therapeutic target for mitigating specific respiratory disease risks and warrants further investigation in clinical trials.

### Supplementary Information

Supplementary Material 1.

Supplementary Material 2.

## Data Availability

The datasets generated and/or analyzed during the current study are publicly available.

## Abbreviations

COPD: Chronic obstructive pulmonary disease
IL6: Interleukin-6
MR: Mendelian randomization
IVs: Instrumental variables
CRP: C-reactive protein
GWAS: Genome-wide association study
IVW: Inverse-variance weighted
MR-PRESSO: MR pleiotropy residual sum and outlier
OR: Odds ratio
CI: Confidence interval
