# Supplementary material for: Genetic perturbation of IL-6 receptor signaling pathway and risk of multiple respiratory diseases
Source: J Transl Med. 2024 Jun 19;22:581. doi: 10.1186/s12967-024-05366-6 (PMC11188576; doi:10.1186/s12967-024-05366-6)
Supplement: Supplementary file 1 — Supplementary Material 1. [file 12967_2024_5366_MOESM1_ESM.docx]

**Table S1.** Genetic variants included for IL6 signaling downregulation.

| **SNP** | **A1** | **A2** | **EAF** | **beta** | **se** | **P** | **R^2^** | **F** |
| --- | --- | --- | --- | --- | --- | --- | --- | --- |
| rs3766925 | A | T | 0.2197 | -0.0148 | 0.0025 | 2.69E-09 | 7.51E-05 | 35.05 |
| rs78035035 | A | C | 0.0138 | 0.0457 | 0.0081 | 1.43E-08 | 5.68E-05 | 31.83 |
| rs112203594 | A | C | 0.0155 | 0.0396 | 0.0071 | 2.09E-08 | 4.79E-05 | 31.11 |
| rs3738028 | A | C | 0.5718 | 0.0137 | 0.0023 | 1.19E-09 | 9.19E-05 | 35.48 |
| rs116141616 | A | G | 0.0170 | 0.0387 | 0.0069 | 1.76E-08 | 5.01E-05 | 31.46 |
| rs12406117 | A | G | 0.5277 | 0.0124 | 0.0021 | 3.83E-09 | 7.66E-05 | 34.87 |
| rs144029367 | T | C | 0.9867 | -0.0498 | 0.0080 | 5.43E-10 | 6.53E-05 | 38.75 |
| rs61806853 | T | C | 0.9662 | 0.0437 | 0.0050 | 1.23E-18 | 1.25E-04 | 76.39 |
| rs76289529 | T | C | 0.0241 | -0.0519 | 0.0060 | 4.78E-18 | 1.27E-04 | 74.82 |
| rs6698385 | A | G | 0.8366 | -0.0350 | 0.0026 | 3.74E-41 | 3.35E-04 | 181.21 |
| rs145262901 | A | G | 0.0108 | -0.0610 | 0.0102 | 2.38E-09 | 7.95E-05 | 35.77 |
| rs145909430 | T | C | 0.9860 | 0.1001 | 0.0082 | 2.86E-34 | 2.76E-04 | 149.02 |
| rs41269913 | T | C | 0.0268 | -0.0424 | 0.0058 | 2.28E-13 | 9.38E-05 | 53.44 |
| rs77994623 | T | C | 0.1374 | 0.0460 | 0.0029 | 1.50E-58 | 5.02E-04 | 251.61 |
| rs183641528 | A | G | 0.0112 | -0.0851 | 0.0080 | 1.62E-26 | 1.60E-04 | 113.16 |
| rs113580743 | A | G | 0.0439 | 0.0550 | 0.0055 | 1.09E-23 | 2.54E-04 | 100.00 |
| rs34693607 | C | G | 0.8453 | 0.0328 | 0.0026 | 3.83E-36 | 2.81E-04 | 159.15 |
| rs56100876 | A | G | 0.0153 | -0.1170 | 0.0086 | 3.27E-42 | 4.12E-04 | 185.09 |
| rs12735458 | A | G | 0.9898 | 0.0842 | 0.0092 | 4.53E-20 | 1.43E-04 | 83.76 |
| rs73026617 | T | C | 0.1055 | 0.0467 | 0.0034 | 1.69E-42 | 4.12E-04 | 188.66 |
| rs7525477 | A | G | 0.3931 | 0.0296 | 0.0023 | 1.35E-38 | 4.18E-04 | 165.63 |
| rs11264224 | A | C | 0.9100 | 0.0418 | 0.0028 | 1.60E-49 | 2.86E-04 | 222.86 |
| rs16836054 | A | G | 0.1266 | 0.0516 | 0.0028 | 1.51E-75 | 5.89E-04 | 339.61 |
| rs12059682 | T | C | 0.7178 | -0.0474 | 0.0025 | 2.11E-77 | 9.10E-04 | 359.48 |
| rs12083537 | A | G | 0.7747 | 0.0679 | 0.0026 | 3.03E- 156 | 1.61E-03 | 682.01 |
| rs2228145 | A | C | 0.6827 | 0.0947 | 0.0021 | 3.00E- 307 | 3.89E-03 | 2033.58 |

F statistic was calculated using the following formulas: R^2^=2×β^2^×EAF×(1-EAF) and F=(beta/se)^2^, where R^2^ represents the phenotypic variance explained by a genetic instrument, F represents F statistic, β is the estimated genetic association of SNP with the exposure, EAF is the effect allele frequency. SNP, single nucleotide polymorphisms.
