# Supplementary material for: Genetic perturbation of IL-6 receptor signaling pathway and risk of multiple respiratory diseases
Source: J Transl Med. 2024 Jun 19;22:581. doi: 10.1186/s12967-024-05366-6 (PMC11188576; doi:10.1186/s12967-024-05366-6)
Supplement: Supplementary file 2 — Supplementary Material 2. [file 12967_2024_5366_MOESM2_ESM.docx]

**Supplementary Figures**

**Figure S1. Scatter plots showing the causal association of IL6R down-regulation on multiple respiratory diseases.**

**Figure S2. Funnel plots for IL6R down-regulation on multiple respiratory diseases.**

**Figure S3. Leave-one-out plots for IL6R down-regulation on lung cancer based on multiple respiratory diseases.**

**Figure S1.** Scatter plots showing the causal association of IL6R down-regulation on multiple respiratory diseases, with the slope of each line corresponding to estimated causal effect per method. A, chronic obstructive pulmonary disease (COPD); B, bronchitis; C, asthma; D, pulmonary embolism; E, idiopathic pulmonary fibrosis; F, lung cancer.

**Figure S2.** Funnel plots for IL6R down-regulation on multiple respiratory diseases. A, chronic obstructive pulmonary disease (COPD); B, bronchitis; C, asthma; D, pulmonary embolism; E, idiopathic pulmonary fibrosis; F, lung cancer.

**Figure S3.** Leave-one-out plots for IL6R down-regulation on lung cancer based on multiple respiratory diseases. A, chronic obstructive pulmonary disease (COPD); B, bronchitis; C, asthma; D, pulmonary embolism; E, idiopathic pulmonary fibrosis; F, lung cancer.
